# Topical Unsaturated Fatty Acid Vesicles Improve Antioxidant Activity of Ammonium Glycyrrhizinate

**DOI:** 10.3390/pharmaceutics13040548

**Published:** 2021-04-14

**Authors:** Maria Chiara Cristiano, Antonia Mancuso, Massimo Fresta, Daniele Torella, Federica De Gaetano, Cinzia Anna Ventura, Donatella Paolino

**Affiliations:** 1 Department of Experimental and Clinical Medicine, University of Catanzaro “Magna Graecia”, Viale Europa s.n.c., 88100 Catanzaro, Italy; mchiara.cristiano@unicz.it (M.C.C.); dtorella@unicz.it (D.T.); 2 Department of Health Sciences, University of Catanzaro “Magna Graecia”, Viale Europa s.n.c., 88100 Catanzaro, Italy; antonia.mancuso@unicz.it (A.M.); fresta@unicz.it (M.F.); 3 Dipartimento di Scienze Chimiche, Biologiche, Farmaceutiche e Ambientali, Università Degli Studi di Messina, Viale Ferdinando Stagno D’Alcontres 31, 98166 Messina, Italy; fedegaetano@unime.it (F.D.G.); caventura@unime.it (C.A.V.)

**Keywords:** unsaturated fatty acids, ammonium glycyrrhizinate, topical delivery, linoleic acid, oleic acid, skin tolerability, antioxidant activity

## Abstract

Linoleic and oleic acids are natural unsaturated fatty acids involved in several biological processes and recently studied as structural components of innovative nanovesicles. The use of natural components in the pharmaceutical field is receiving growing attention from the scientific world. The aim of this research work is to design, to perform physico-chemical characterization and in vitro/in vivo studies of unsaturated fatty acids vesicles containing ammonium glycyrrhizinate, obtaining a new topical drug delivery system. The chosen active substance is well known as an anti-inflammatory compound, but its antioxidant activity is also noteworthy. In this way, the obtained nanocarriers are totally natural vesicles and they have shown to have suitable physico-chemical features for topical administration. Moreover, the proposed nanocarriers have proven their ability to improve the in vitro percutaneous permeation and antioxidant activity of ammonium glycyrrhizinate on human keratinocytes (NCTC 2544 cells). In vivo studies, carried out on human volunteers, have demonstrated the biocompatibility of unsaturated fatty acid vesicles toward skin tissue, indicating a possible clinical application of unsaturated fatty acid vesicles for the treatment of topical diseases.

## 1. Introduction

The topical administration of active compounds remains little explored due to the poor availability of effective drug delivery systems, despite the progress of nanomedicine. The main limit of topical permeation of drug is represented by the formidable barrier of the stratum corneum, which limits the cutaneous passage of many drugs. For this reason, several strategies were proposed to enhance skin penetration, involving physical methods such as iontophoresis, electroporation, phonophoresis, and microneedles, as a single or combined application [1]. Despite the effectiveness of these methods being widely demonstrated [2,3,4,5], their application is limited by the resulting skin irritation and by their invasive character, even if minimal. For example, Curdy et al. [6] demonstrated that a higher intensity of current used during iontophoresis can cause mild to moderate skin damage on the application site of the patch as a function of exposure time. Other minor reactions, i.e., erythema, itching, and tingling, are common following a physical method of drug delivery [7]. As an alternative to the cited methods, new vesicular systems were developed, such as liposomes, ethosomes^®^, transfersomes^®^, niosomes etc., in order to ensure adequate penetration across the skin [8]. The first ones are characterized by a peculiar rigidity of their lipid bilayer, which limits their application as topical drug delivery systems [9]. On the contrary, ethosomes^®^ and transfersomes^®^ are characterized by a correct deformability, which makes them able to interact with skin structures and cross the stratum corneum [10,11]. These nanosystems are also promising for topical application because they have demonstrated good safety profiles during in vivo and in vitro studies [12]. Moreover, in the last few years, researchers have overcome the problem of scaling up the nanosystems by means of microfluidic methods [13]. Starting from the features that make ethosomes^®^ and transfersomes^®^ ideal nanocarriers for topical delivery, we wanted to evaluate the potential of unsaturated fatty acid vesicles to increase percutaneous permeation of active compounds.

Linoleic and oleic acids are unsaturated fatty acids obtained from natural sources involved in several biological processes. Several research groups have demonstrated the ability of linoleic and oleic acids to induce a lightening effect on the skin [14]. For example, Ando et al. demonstrated by means of in vitro experiments that the lightening effect is a consequence of the inhibition of melanin production in melanocytes, and this inhibition seems to be related to the number of unsaturated bonds and their position. In this way, the authors explained the greater whitening activity of linoleic acid compared with oleic acid, characterized by two and a single unsaturated bond, respectively [14]. For this reason, several drug delivery systems were used to deliver linoleic acid and to improve the biological effect of this unsaturated fatty acid [15,16,17]. The benefits induced by linoleic acid on skin were known for many years. For example, Skolnik et al. [18] demonstrated that the topical application of linoleic acid, after its percutaneous absorption, was sufficient to induce a reduction of the cutaneous signs in EFA (essential fatty acid)-deficient patients. EFA-deficiency was identified for the first time in rodants, and only later in men; the EFA-deficient patients are characterized by low weight gain and skin abnormalities, especially if the disease occurs in newborns [19,20,21]. As well as Skolnik, other research groups demonstrated the beneficial effects related to topical application of sunflower-seed oil on patients affected by essential fatty acid deficiency [19,20,21].

Regarding oleic acid and its effects on the skin, the literature highlights the ability of oleic acid to act as a penetration enhancer. For this reason, some vesicles made up of this unsaturated fatty acid have already been developed [22,23]. Oleic acid, thanks to its single unsaturated bond, is able to induce a selective perturbation of the extra cellular lipid bilayer in the stratum corneum. This temporary disorganization, similar to ethanol contained in ethosomes [11,12], was exploited by Torchilin et al. [24] for inducing increased skin permeability and the release of fluorescein entrapped into liposomes based on oleic acid. Other research groups have demonstrated the effects of oleic acid on endogenous stratum corneum lipids. The insertion of this unsaturated fatty acid in the bilayer seems to induce a perturbation in skin and, so, a fluidizing effect [25,26].

Previously, we combined the presence of linoleic acid and oleic acid in a unique nanosystem [27], obtaining promising results in terms of oleuropein delivery for gastrointestinal use. To the best of our knowledge, no studies on the topical application of unsaturated fatty acid vesicles made up of both linoleic and oleic acids were carried out. Thus, the aim of this work was to combine the skin effects of both unsaturated fatty acids for the design of a new totally natural topical drug delivery system. The coexistence of oleic acid and linoleic acid in forming a single delivery system could be useful in the topical delivery of active molecules for the treatment of skin diseases. Since oleic acid acts as a penetration enhancer, its action could improve the penetration through the stratum corneum of vesicles. The skin barrier repairing properties of linoleic acid are well known, related to increased keratinocyte proliferation and lipid synthesis [28,29]. Therefore, it is our opinion that the combination of the two unsaturated fatty acids could provide a topical delivery system capable by itself of inducing an improvement in the pathological condition of the skin.

The obtained vesicles were used and tested for the delivery of ammonium glycyrrhizinate, an endemic natural compound derived from *glycyrrhiza glabra*, abundant in our territory (Calabria, Italy), as well as in Spain, Greece, and in the entire Mediterranean area. The potential of ammonium glycyrrhizinate has been amply demonstrated for many pathologies and skin disease [30], as an anti-inflammatory and antioxidant compound [31,32,33]. The topical administration of ammonium glycyrrhizinate is severely limited by its physico-chemical properties and in particular by its high molecular weight and amphiphilic structure. These properties are poorly compatible with a passive diffusion through the skin. On these bases, the use of a nanosystem that transport the drug through skin is needed. In this paper, ammonium glycyrrhizinate-loaded unsaturated fatty acid vesicles were prepared and characterized, in order to be proposed as innovative tools in therapeutic treatments of oxidative stress in skin disease.

## 2. Materials and Methods

### 2.1. Material

All substances and solvents used in this experimental work were highly pure and did not require further purification procedures. In detail, phospholipon^®^ 90G (PL90G) was obtained from Lucas Meyer C., Hamburg, Germany. Oleic acid, linoleic acid, ammonium glycyrrhizinate, 3-(4,5-dimethylthiazol-2-yl)-2,5-diphenyl-*2H*-tetrazolium bromide (MTT), and ethanol were purchased from Sigma-Aldrich (Sigma-Aldrich, Darmstadt, Germany). For release analysis, Spectra/Por cellulose membranes, with a cut-off of 10,000 Da, were used and they were purchased from Spectrum Laboratories Inc (Rancho Dominguez, CA, USA). NCTC2544 cells were provided by the Instituto Zooprofilattico of Modena and Reggio Emilia (Instituto Zooprofilattico of Modena and Reggio Emilia, Reggio Emilia, Italy); all reagents necessary for in vitro studies, such as trypsine/EDTA, culture medium (DMEM) and fetal bovine serum (FBS) were obtained from GIBCO (Invitrogen Corporation, Paisley, UK). Finally, double distilled water was used to prepare all the samples.

### 2.2. Preparation and Physico-Chemical Characterization of Unsaturated Fatty Acid Vesicles

The preparation method of unsaturated fatty acid vesicles was designed and already described by our research group [27]. Briefly, three batches of formulation A and formulation B were obtained by mixing oleic acid, linoleic acid, and PL90G (total lipid component concentration equal to 6.67 mg/mL) with an appropriate molar ratio (Table 1). PL90G is defined as lecithin and was included in the formulation for its ability to act as an emulsifier and suspension stabilizer [34]. The obtained lipid mixtures were suspended in double distilled water (pH = 7.4) and then homogenized at 15,000 rpm for 20 min using an Ultra-Turrax T25 (IKA®-Werke GmbH & Co. KG, Staufen, Germany). To prepare ammonium glycyrrhizinate-loaded unsaturated fatty acid vesicles, the natural compound (3 mg/mL) [35,36] was added in the lipid components during the preparation of the nanosystems.

The physico-chemical characterization of unsaturated fatty acid vesicles was carried out by dynamic light scattering using the Zetasizer Nano ZS (Malvern Instruments, Malvern, UK), determining average sizes, polydispersity index (PdI), and Z-potential values. All samples were diluted (1:20 vesicular suspension:double-distilled water) to avoid multi-scattering phenomena. The Zetasizer Nano ZS is a spectrophotometer equipped with a rated output of 4.5 mW laser diode, operating at 670 nm with a backscattering angle of 173°. Fundamental parameters to obtain average sizes and PdI values were set before each analysis, and in detail the dielectric constant, the medium refractive index and the medium viscosity were set, respectively, at 80.4, 1.330, and 1.0 mPa·s. Net surface charge values were measured by electrophoretic mobility using a Smoluchowsky constant F (Ka) of 1.5, as a function of the electrophoretic mobility of the nanosystems [37]. Each analysis was performed using three different aliquots of the same batch (three batches were prepared for each formulation).

### 2.3. Deformability Index Evaluation

The deformability Index (DI) of unsaturated fatty acid vesicles was evaluated as already described by Manca et al. [38]. Briefly, the nanosystems were extruded through a 50 nm pore size polycarbonate membrane filters at a constant pressure of 2.5 bar for 10 min, using a Lipex Biomembranes extruder (Northern Lipids Inc., Vancouver, BC, Canada). The values of DI were obtained applying the following Equation (1):

(1) $$\mathrm{DI}=\left[\;J\;\left(\frac{d0}{p}\right)\left(\frac{d0}{d0-d1}\right)\right]$$

where J is the ratio between the weight of unsaturated fatty acid vesicles aliquot before and then extrusion; d0 and d1 are the mean size of vesicles before and then extrusion, respectively; and p is the pore size of membrane filters. Three batches were prepared for each formulation and three different analyses were performed considering three rates deriving from the same batch.

### 2.4. Entrapment Efficacy and Drug Loading Capability of Unsaturated Fatty acid Vesicles

The entrapment efficacy (EE%) of ammonium glycyrrhizinate in purified formulation A and B was evaluated by using Amicon Ultra 3000 Da MWCO-Merck Millipore (Merck Millipore, Molsheim, France) [27]. The samples were centrifuged at 4000 rpm at room temperature for 20 min in a centrifuge Eppendorf 5810. The obtained pellet was divided from supernatant and it was solubilized by using ethanol (99.9% purity). Ammonium glycyrrhizinate, contained in the supernatant as unentrapped active substance and in solubilized pellet as entrapped one, was detected by using HPLC (High Performance Liquid Chromatography) (see Section 2.7). The entrapment efficacy (EE%) and drug loading (DL%) were calculated using the following Equations (2) and (3):

(2) $$\mathrm{EE}\%=\frac{\mathrm{De}}{\mathrm{De}+\mathrm{Ds}}\times 100$$

(3) $$\mathrm{DL}\%=\frac{\mathrm{De}}{\mathrm{Lw}}\times 100$$

where De and Ds are the amounts of ammonium glycyrrhizinate detected in the pellet and in the supernatant, respectively, and Lw represents the amount of lipid component used to make vesicles [39]. The results are expressed as the mean value of three different experiments performed on each realized batch (*n* = 3) ± standard deviation.

### 2.5. In Vitro Release Evaluation

The in vitro release profiles of ammonium glycyrrhizinate from formulation A and formulation B were obtained by using dynamic Franz diffusion cells (LGA, Berkeley, CA, USA). These skin permeation systems, characterized by a diffusional surface area of 0.75 cm^2^ and a volume of 4.75 mL, were composed by a donor and a receptor compartment. For in vitro release studies, a synthetic cellulose membrane (molecular cut-off 10.000 Da) was placed between the two compartments. The donor compartment hosted the two formulations (200 µL) to be tested or a hydroalcoholic solution of free ammonium glycyrrhizinate (200 µL; 3 mg/mL); while the receptor compartment was filled with a hydroalcoholic solution (70:30 double-distilled water:ethanol) and was maintained under continuous stirring to have sink conditions during the experiments [40]. At pre-fixed time intervals (up to 72 h), 200 µL of samples were collected from the receptor compartment and immediately replaced with fresh hydroalcoholic solution. The amount of ammonium glycyrrhizinate released at the different incubation time was quantified by using HPLC (see Section 2.7). The results are expressed as the mean value of three different experiments performed on each realized batch (*n* = 3) ± standard deviation.

### 2.6. In Vitro Percutaneous Permeation of Ammonium Glycyrrhizinate-Loaded Unsaturated Fatty Acid Vesicles

Dynamic Franz diffusion cells were also used for evaluating the in vitro percutaneous permeation of ammonium glycyrrhizinate in free form and in unsaturated fatty acid vesicles through human stratum corneum and viable epidermis (SCE) membranes. In detail, human skin sections were obtained from abdominal reduction surgery of human patients (age 50 ± 2 years). According to the method previously described by Kligman and Christophers [41], the subcutaneous fat was gently removed, and skin samples were put in distilled water at 60 ± 1 °C for 2 min. SCE membranes were isolated from dermis and they were dried in a desiccator and then stored at 4 ± 1 °C until use. Before the in vitro percutaneous permeation studies, tritiated water was used to evaluate the integrity of skin section and a permeation coefficient value of 1.2 ± 0.2 × 10^−3^ cm/h was obtained and it was considered acceptable for guaranteeing the skin integrity [42].

For in vitro percutaneous permeation studies, SCE membranes were interposed between the receptor and the donor compartments of Franz diffusion cell, with the stratum corneum side up and after their hydration in isotonic sterile saline solution. The experimental conditions were the same of in vitro release studies (see Section 2.5.). The donor was filled with 200 µL of each formulation, while receptor was filled with a hydroalcoholic solution (70:30 double-distilled water:ethanol) and at pre-fixed intervals aliquots of the receptor solution were withdrawn and immediately replaced with fresh hydroalcoholic solution. The collected aliquots were analyzed by HPLC (see Section 2.7.). The temperature was maintained at 32.0 ± 0.5 °C for the entire experiments (24 h) by means of a circulating water bath. The results are expressed as the mean value of three different experiments for each realized batch (*n* = 3) ± standard deviation.

### 2.7. HPLC (High Performance Liquid Chromatography) Analysis

The samples of ammonium glycyrrhizinate derived from entrapment efficacy determination, in vitro release and in vitro percutaneous permeation studies, were analyzed using HPLC (A Jasco PU-1580 intelligent HPLC pump, Tokyo, Japan). Chromatographic conditions for reversed-phase HPLC with UV (ultraviolet) photodiode array detection were as follows: column, Phenomenex Jupiter C18, 5 µm; column temperature, 25 °C; mobile phase, a 30:60:10 (*v*/*v* ratio) mixture of acetonitrile:methanol:phosphoric acid (0.1 N); flow rate, 1 mL/min. The UV detection wavelength was 247 nm [35,36] (accuracy was 99.76%).

### 2.8. Cell Culture

The eventual cytotoxic effects of unsaturated fatty acid vesicles and antioxidant activity of ammonium glycyrrhizinate-loaded nanosystems were evaluated on human keratinocytes (NCTC 2544 cell line). Cells were incubated in plastic culture dishes (100 × 20 mm) in a water jacketed incubator (Thermo Fisher Scientific, Rome, Italy) at 37 °C and 5% CO_2_, in presence of minimum essential medium (D-MEM) enriched with glutamax, streptomycin (100 µg/mL)-penicillin (100 µL/mL) solution (1% *v*/*v*), amphotericin B (250 µg/mL), and FBS (10% *v*/*v*). For cell maintenance, the culture medium was replaced with a fresh medium every 48 h, until ~80% cellular confluence was achieved. The cells were washed with phosphate buffer solution (PBS) and were treated with trypsin/EDTA (1×) solution to induce their detachment. The detached cells were collected in a centrifuge tube and centrifuged (1000 rpm) at room temperature for 10 min with an Eppendorf Centrifuge 5810. Finally, before in vitro experiments, a fresh D-MEM medium was used to resuspend the pellet.

### 2.9. In Vitro Evaluation of Protective Effect Induced by Ammonium Glycyrrhizinate-Loaded Unsaturated Fatty Acid Vesicles: MTT and LDH Assay

The protective effects of ammonium glycyrrhizinate-loaded unsaturated fatty acid vesicles were evaluated on NCTC 2544 cells by means MTT test and LDH assay after 24 h of treatments and the results were expressed as cell viability (%) and LDH release (%), respectively. For in vitro assay, NCTC 2544 cells were placed in 96-well culture plate at a density of 5000 cells/0.2 mL, and after 24h of incubation, the cells were treated with ammonium glycyrrhizinate (0.03 mM) in free form and entrapped in formulation A and formulation B for 24 h, and then with H_2_O_2_ (700 µM) for 1 h. The concentration and incubation time of H_2_O_2_ were chosen based on preliminary experiments (data not shown).

For the MTT test, the cells were additionally treated with empty Formulation A and Formulation B to evaluate the potential cytotoxic effects induced by unsaturated fatty acid vesicles. At the end of treatments, 10 μL of MTT (5 mg/mL dissolved in PBS solution) was placed in each well; after 3 h of incubation, supernatants were removed and (200 μL) dimethyl sulfoxide/ethanol solution (1:1 *v*/*v*) was added to each well to solubilize the colored formazan crystals. The plates were gently shaken at 230 rpm (IKA^®^ KS 130 Control, IKA^®^ WERKE GMBH & Co, Staufen, Germany) for 20 min. The ELISA microplate reader xMark™ Microplate Absorbance Spectrophotometer (Bio-Rad Laboratories, Milan, Italy) was used to study the absorbance values of all the analyzed samples at a wavelength of 540 nm and a reference wavelength of 690 nm. Cell viability was calculated according to the following Equation (4):

(4) $$\mathrm{Cell}\;\mathrm{viability}\;\left(\%\right)=\frac{\mathrm{AbsT}}{\mathrm{AbsC}}\times 100,$$

where AbsT is the absorbance of treated cells and AbsC is the absorbance of untreated cells used as control.

The antioxidant activity of ammonium glycyrrhizinate in free form or entrapped into unsaturated fatty acid vesicles was evaluated by measuring the lactic hydrogenase (LDH) release (%), as a function of cellular membrane alteration and cell disruption [43], after a chemical solicitation carried out by means the treatment with hydrogen peroxide (H_2_O_2_). As for the MTT test, during the LDH assay, the obtained results were compared to the effects induced by empty unsaturated fatty acid vesicles.

A Pierce LDH cytotoxic assay kit and a spectrophotometer (xMark™ Microplate 483 Absorbance Spectrophotometer, Bio-Rad Laboratories, Milan, Italy) were used to quantify the LDH release, as indication of membrane damage. The spectrophotometer, as indicated in the protocol of Pierce LDH cytotoxic assay kit, was set at λ = 680 nm and λ = 490 nm.

The percentage of LDH release was obtained from the following Equation (5):

(5) $$\%\mathrm{LDH}\;\mathrm{release}=\frac{{\mathrm{Compound}}_{\mathrm{treated}}\mathrm{LDH}\;\mathrm{activity}-\mathrm{Spontaneous}\;\mathrm{LDH}\;\mathrm{activity}}{\mathrm{Maximum}\;\mathrm{LDH}\;\mathrm{activity}-\mathrm{Spontaneous}\;\mathrm{LDH}\;\mathrm{activity}}\times 100,$$

where Compound treated LDH activity and Spontaneous LDH activity correspond to the LDH released by treated cells and untreated cells, respectively; Maximum LDH activity corresponds to the maximum LDH release obtained from lysed cells. The values of LDH activity for each sample was obtained subtracting the 680 nm absorbance value (background) from the 490 nm absorbance, before calculation of %LDH release. The results were reported as the average of three independent experiments ± standard deviation.

### 2.10. Skin Tolerability Evaluation of Unsaturated Fatty Acid Vesicles on Human Volunteers

In vivo tolerability of unsaturated fatty acid vesicles was evaluated in terms of erythema index variation, by using a non-invasive reflectance spectrophotometry SP60 (X-Rite Incorporated, Grandville, MI, USA). The spectrophotometer was characterized by a 0° illumination and a 45° viewing angle and it was properly calibrated [35]. Reflectance spectra were obtained over the wavelength range 400–700 nm by using illuminant C and 28 standard observers.

For in vivo studies, human volunteers (*n* = 20), belonging to both sexes and aged between 22 and 28 years, were recruited. Before starting the investigation, the volunteers were informed about the nature of the study, signed a consent form, and they rested at room condition for 30 min before the experiments.

Six sites (two for each tested formulation) on the ventral surface of each forearm were chosen and defined using a template (about 1 cm^2^); at least 2 cm of distance between the different sites were left, to avoid interferences. Before to apply on human volunteers’ forearms the formulations, erythema index (EI) baseline values were recorded for each site, and then saline solution (NaCl 0.9% (*w*/*v*) was used as control), formulation A and formulation B (100 µL) were applied on two sites using Hill Top chamber (Hill Top Research, Inc. Cincinnati, OH, USA). After fixed interval time (24, 48 and 72 h), the chambers were removed, and the skin sites were washed with water. The reflectance spectrophotometer was used to monitor possible induced erythema. Erythema index values were obtained according to the following Equation (6):

(6) $$\mathrm{EI}=100\;\left[\log\log\;\frac{1}{R560}+1.5\;\left(\log\log\;\frac{1}{R540}+\log\log\;\frac{1}{R580}\right)-2\;\left(\log\log\;\frac{1}{R510}+\;\log\log\;\frac{1}{R610}\right)\right]$$

where R is the reflectance value at a specific wavelength (560, 540, 580, 510 and 610). The results are expressed as ΔEI, obtained from the difference between the EI value measured after each treatment and the EI baseline value measured before the treatments at each application site.

### 2.11. Statistical Analysis

One-way ANOVA test was used for statistical data analysis. Bonferroni *t*-test was used to confirm the obtained results. A * *p* value < 0. 05 and a ** *p* value < 0.001 were considered statistically significant and highly statistically significant, respectively.

## 3. Results and Discussion

### 3.1. Physico-Chemical Characterization of Ammonium Glycyrrhizinate-Loaded and Blank Unsaturated Fatty Acid Vesicles

The physico-chemical characteristics that drug delivery systems must have in order to be effectively applied to the skin were well defined. In detail, a topical nanosystem should be biocompatible, non-irritative, having good penetration properties, and above all it should have specific properties in terms of average size, size distribution and surface charge [44,45]. These properties guarantee an effective interaction of nanosystems with skin structure and their passage through stratum corneum.

Previously, other research groups investigated the behavior of unsaturated fatty acid when they were dispersed in water at neutral pH [46,47,48]. In detail, it was demonstrated that the precipitation of fatty acid or the formation of micelles were dependent on pH. In fact, in presence of acid pH, the carboxylate groups of unsaturated fatty acid are protonated inducing the precipitation; while at basic pH carboxylate groups are charged and induce a spontaneous formation of micelles. For this reason, the pH at which it operates must necessarily be close to neutrality in order to ensure the formation of functional vesicles. In presence of neutral pH, unsaturated fatty acids such as oleic acid, assemble in bilayer to form vesicles characterized by specific physico-chemical features as a function of their composition [49].

In this research work, two different formulations were prepared and characterized. As shown in Table 2, unsaturated fatty acid vesicles made up of only unsaturated fatty acid (formulation B) were characterized by a hydrodynamic diameter greater respect to formulation A. Probably the presence of lecithin (PL90G) in the formulation A induced a compaction of the vesicular structure of the nanosystems [34], reducing the average size. An effective topical application requires a reduced mean size and a narrow size distribution and, observing the values reported in Table 2, we can affirm that ammonium glycyrrhizinate made the nanosystems more suitable for topical application. The mean size, particularly for formulation B, underwent a marked reduction, returning to a dimensional range considered suitable for the application on the skin [50]. Since ammonium glycyrrhizinate is an amphiphilic compound, it was probably collocated both between lipid bilayer and aqueous core, and between the lipid bilayer and external aqueous environment outside the vesicle. This would explain not only the high encapsulation efficiency (EE > 80% for both formulations) but also the increase in the net negative surface charge in ammonium glycyrrhizinate-loaded unsaturated fatty acid vesicles [36]. Moreover, values greater than 35% of drug loading capability were recorded for both samples containing ammonium glycyrrhizinate. The presence of ammonium glycyrrhizinate within unsaturated fatty acid did not modify the stability and the homogeneity of nanosystems, as confirmed by PdI values. A low polydispersity index value means a tight dimensional distribution and a firm interaction of the active compound in the structure of the system. The encapsulation of ammonium glycyrrhizinate in both formulations and the persistence of good characteristics in terms of average size, PdI, net surface charge values, even in the presence of the active substance, are not in line with previously obtained data about oleuropein-loaded ufasomes [27]. These different trends highlighted how the same vesicular system may or may not be suitable for the delivery of an active ingredient depending on the chemical-physical characteristics of the chosen compound.

The choice of using linoleic acid and oleic acid for the constitution of unsaturated fatty acid vesicles was borne from the peculiar characteristics of the two fatty acids, and from their easy availability. In particular, several research groups have demonstrated that the presence of oleic acid and/or linoleic acid induce an increased deformability of nanosystems [51,52,53]. Since deformability is a key feature for guaranteeing lipid vesicles penetration through the skin, in this study, the prepared formulations were subjected to deformability assay by the extrusion method, and the obtained results were compared to already published data. Unsaturated fatty acid vesicles showed values of DI equal to 9.71 ± 0.87 and 12.73 ± 1.01 for formulation A and B, respectively (Table 2). These DI values were considered good values when compared with transfersomes, which are considered as ultradeformable vesicles made up by phospholipids and edge activators [36,54,55], or with liposomes that are considered unsuitable for topical delivery due to their particularly rigid bilayer [56]. The demonstrated deformability of unsaturated fatty acid vesicles was due to the presence of *cis* double bonds in hydrocarbon chain of oleic and linoleic acid [26], that make more fluid the bilayer. The role of oleic acid in inducing an increase in the deformability of the vesicles has also been demonstrated by Song et al. [51]. Moreover, we can affirm that the presence of ammonium glycyrrhizinate in the unsaturated fatty acid vesicles did not negatively influence the deformability.

### 3.2. In Vitro Release Profile and Percutaneous Permeation Profile of Ammonium Glycyrrhizinate-Loaded Unsaturated Fatty Acid

Following entrapment efficacy evaluation, the ability of Formulation A and B to release ammonium glycyrrhizinate was evaluated by using dynamic Franz diffusion cells and a synthetic membrane.

**Figure 1:**
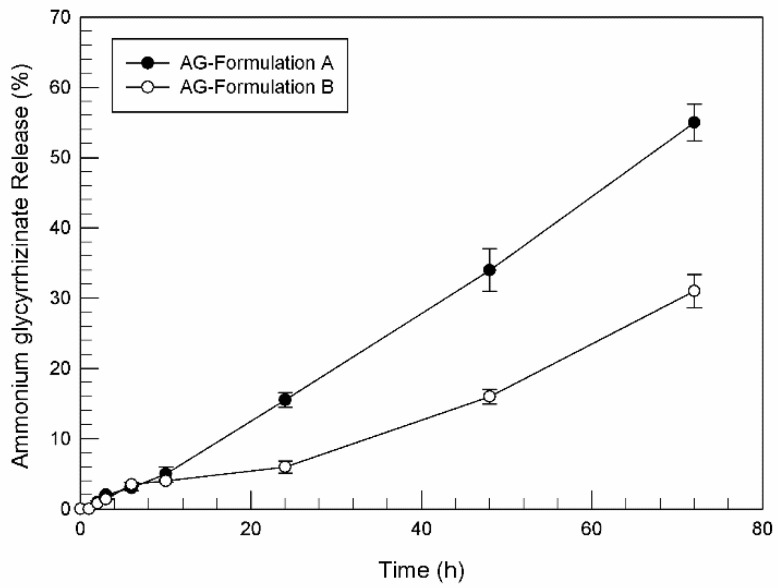
Release profile (%) of ammonium glycyrrhizinate-loaded Formulation A and Formulation B, as a function of time. Experiments were carried out using dynamic diffusion Franz cells and synthetic membrane, and the results were expressed as mean values of three different experiments from three different batches ± standard deviation. If bars are not visible, they are within the symbol.

As shown in Figure 1, quite unexpectedly, the release of ammonium glycyrrhizinate from tested unsaturated fatty acid vesicles resulted very low, above all within the first 24 h, reaching 15.5% (±1.0) and 6% (±0.8) of released ammonium glycyrrhizinate for formulation A and formulation B, respectively, while 50% of the released compound was detected only after 72 h of analysis and only for formulation A. Since the amount of encapsulated ammonium glycyrrhizinate was similar for both formulations, certainly the different release profile depends on the chemical-physical composition of carriers. Probably, the presence of PL90G^®^ in formulation A induced a rearrangement of the bilayer such as to favor an easier release of the compound.

**Figure 2:**
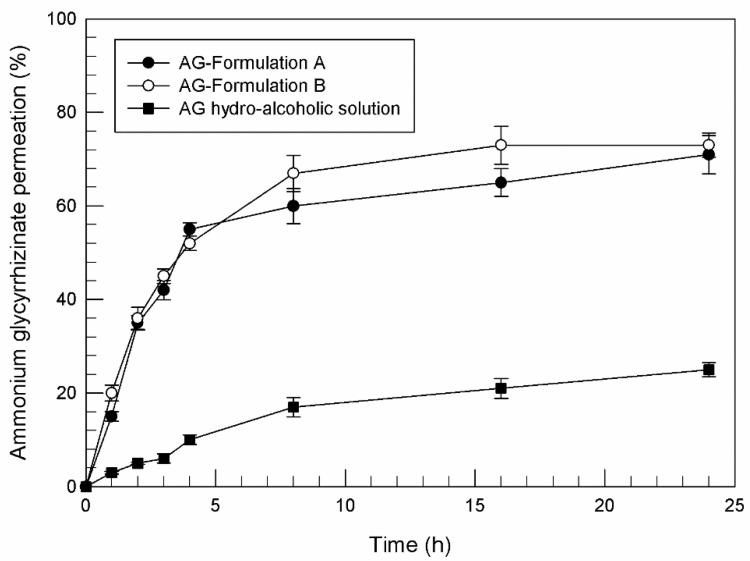
Percutaneous permeation profile (%) of ammonium glycyrrhizinate-loaded unsaturated fatty acid vesicles and ammonium glycyrrhizinate hydroalcoholic solution, obtained by using dynamic diffusion Franz cells and human stratum corneum and epidermis (SCE) membranes. Results are expressed as mean values of three different experiments from three different batches ± standard deviation. If bars are not visible, they are within the symbol. All data related to AG-formulation A and B are statistically significant with respect to AG hydroalcoholic solution data (*p* < 0.001). (AG = ammonium glycyrrhizinate).

On the contrary, the profiles related to percutaneous permeation of ammonium glycyrrhizinate-loaded unsaturated fatty acid vesicles were satisfying (Figure 2). For this investigation, human SCE membranes were used as interposed between the two compartments of dynamic diffusion Franz cells. As shown in Figure 2, the ability of ammonium glycyrrhizinate in free form (as hydroalcoholic solution at concentration of 2.55 mg/mL that corresponds to the maximum concentration of active substance entrapped in formulation B) to permeate through the stratum corneum was very poor; in fact only 24.8% (±1.54) of the applied active compound was detected in the receptor compartment after 24 h. Indeed, this natural compound is characterized by a high molecular weight and a particular structure that limits its spontaneous permeation through the skin. Thus, to exert its action following topical administration, ammonium glycyrrhizinate needs a suitable system able to improve its passage through skin.

Oleic and linoleic acid vesicles, instead, have demonstrated their ability to improve the passage of entrapped compound through stratum corneum, thanks to their lipid composition. In fact, some unsaturated fatty acids are well known as penetration enhancers in pharmaceutical products [25,57]. In particular, the increased skin permeability induced by oleic and linoleic acid seems to be related to a perturbation of the lipid structures of the stratum corneum, inducing a disorder in the highly packed stratum corneum intercellular domain lipids [58].

Thanks to the active interaction between oleic and linoleic acid with skin structures, the improved ammonium glycyrrhizinate percutaneous permeation in the presence of unsaturated fatty acid vesicles is evident in Figure 2. After 24 h of experiments, 71% (±4.08) and 73% (±2.59) of the entrapped compound in Formulation A and Formulation B, respectively, were permeated through SCE membranes. The difference between unsaturated fatty acid vesicles and hydro-alcoholic solution in terms of induced ammonium glycyrrhizinate permeation is even more evident when comparing the values of fluxes (μg/cm^2^ h^−1^), that permit us to evaluate the quantitative percutaneous permeation through SCE membranes. In detail, the calculated fluxes of ammonium glycyrrhizinate were 15.0 ± 4.9 μg/cm^2^ h^−1^, 16.6 ± 5.2 μg/cm^2^ h^−1^, and 8.1 ± 1.1 μg/cm^2^ h^−1^, respectively for Formulation A, Formulation B and hydro-alcoholic solution.

Comparing the results reported in Figure 1 and Figure 2, and since the same formulations and the same ammonium glycyrrhizinate concentrations were investigated, it is evident that synthetic membranes for diffusion Franz cells are not always suitable to mimic human percutaneous absorption. Based on the physico-chemical characteristics of the tested drugs and tested nanovesicles, biological or synthetic membranes should be adequately chosen in laboratory diffusion tests. The use of biological membranes should be more stimulated, though the commercial availability, stability, interbatch uniformity, and ease of use make synthetic media highly desirable for some studies [59].

### 3.3. In Vitro Antioxidant Effects of Ammonium Glycyrrhizinate-Loaded Unsaturated Fatty Acid Vesicles on NCTC 2544 Cells

Ammonium glycyrrhizinate is well known for its anti-inflammatory [60] and antioxidant activity [61,62]. To deliver this natural compound, improving its pharmacological activity, we chose a totally natural drug delivery system. To evaluate the suitability of the carriers, we tested the effects of ammonium glycyrrhizinate-loaded unsaturated fatty acid vesicles on human keratinocytes in terms of cell viability and LDH release before and after oxidative stress. Moreover, the obtained results were compared with possible in vitro effects induced by empty unsaturated fatty acid vesicles.

To evaluate the protective effects induced by ammonium glycyrrhizinate in free form and as active compound-loaded vesicles, the cells were exposed to H_2_O_2_. Hydrogen peroxide can produce an oxidative stress in biological systems, and even if it is not very reactive, it can sometimes be toxic to cells. In detail, H_2_O_2_ can attack many cellular energy-producing systems and induce an alteration of the cellular membranes [63].

Based on preliminary in vitro studies, carried out testing increasing concentration of ammonium glycyrrhizinate on NCTC 2544 cells, we have chosen to use the concentration of 0.03 mM, because it was found to be the lowest effective concentration (data not shown).

**Figure 3:**
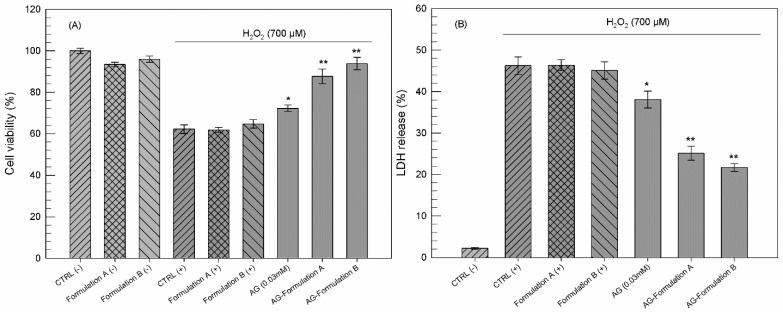
Antioxidant activity of ammonium glycyrrhizinate in free form and loaded in unsaturated fatty acid vesicles, expressed as NCTC 2544 viability (**A**) and lactic hydrogenase (LDH) release (**B**). Cells were treated with ammonium glycyrrhizinate (0.03 mM), alone or loaded in the vesicles for 24 h and then with H_2_O_2_ (700 µM) for 2 h. Results are expressed as mean values of three different experiments from three different batches ± standard deviation. If bars are not visible, they are within the symbol. * *p* < 0.05 and ** *p* < 0.001 versus H_2_O_2_ positive control (CTRL (+)). (AG = ammonium glycyrrhizinate).

As reported in Figure 3, empty unsaturated fatty acid vesicles have been shown to have a good safety profile. We chose to test the amount of vesicles (lipid concentration) equal to that necessary for specific AG concentration delivery. Without oxidative stress, treated cell viability was maintained over 90% (Figure 3A) (Formulation A (-) and Formulation B (-) in the figure). On the other hand, linoleic and oleic acids included into vesicles were unable to exert a significant antioxidant effect at the used concentrations. Indeed, as reported by Figure 3B, blank Formulation A (+) and Formulation B (+) did not significantly influence the lactic dehydrogenase (LDH) release induced by H_2_O_2_ treatments. These results highlighted the safe profile of empty vesicles and, above all, their inertia in biological processes.

Ammonium glycyrrhizinate alone was able to induce a reduction of LDH release less than 10% compared to control cells (CTRL (+)) treated with hydrogen peroxide. This antioxidant effect could be due to the already demonstrated action mechanism of this natural compound. Some research groups have demonstrated the ability of licorice extracts, such as glycyrrhizinate, to act as radical scavengers [62,64].

This protective effect induced by ammonium glycyrrhizinate is enhanced by its encapsulation in unsaturated fatty acid vesicles. As reported in Figure 3, ammonium glycyrrhizinate-loaded Formulation A and Formulation B (AG-Formulation A and B, respectively) were shown to protect cells from induced oxidation compared with CTRL (+); indeed, the LDH release was reduced by about 21% and about 25% from Formulation A and Formulation B, respectively. These encouraging results are probably a consequence of the greater interaction of vesicles and cellular membrane, as already demonstrated by our research group for the same nanocarriers but in another cell line [27]. The cell–vesicles interaction has allowed a greater internalization of ammonium glycyrrhizinate within NCTC 2544 cells, making itself immediately available to protect cells from the subsequent induced oxidative stress.

The improved antioxidant and protective effects of ammonium glycyrrhizinate-loaded unsaturated fatty acid vesicles also affect post oxidative stressed cell viability, as shown in Figure 3A. In detail, when cells were pre-treated with natural compound-loaded vesicles, their viability remained over 80% despite the post treatment with hydrogen peroxide.

Therefore, on the basis of these in vitro results, we can conclude that thanks to the delivery of the ammonium glycyrrhizinate in nanovesicles, the cells retain their viability even in the presence of stress.

### 3.4. In Vivo Skin Tolerability Evaluation

Having ascertained the in vitro safety of unsaturated fatty acid vesicles and their ability to improve percutaneous permeation of the natural compound, in this first part of the research work, we wanted to evaluate the effects of empty vesicles on human skin. For this reason, the skin tolerability of empty vesicles was tested on human volunteers and compared to a negative control, a saline solution. The skin tolerability was evaluated using a reflectance spectrophotometry, a noninvasive technique that evaluates the variation of erythema index as sign of skin color alteration.

**Figure 4:**
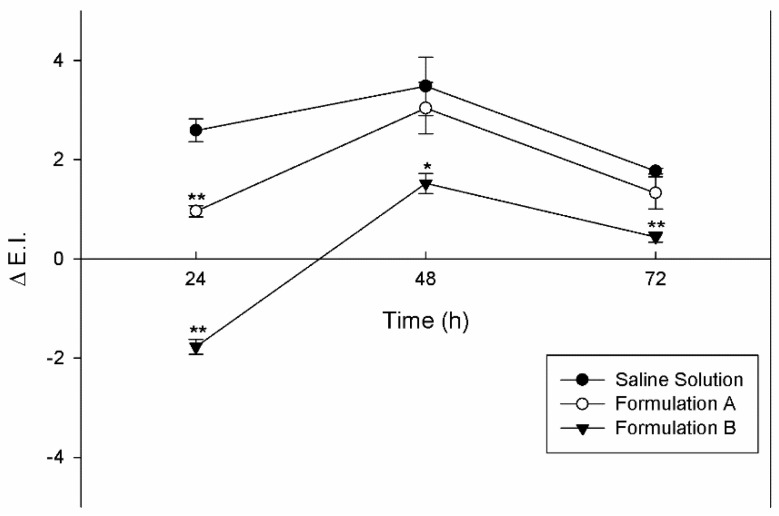
In vivo skin tolerability analysis carried out on human volunteers. Saline solution, Formulation A, and Formulation B were applied on forearms and the Δ E.I. was detected as a function of exposure time (24, 48 and 72h). Results are expressed as mean value of three different experiments from three different batches ± standard deviation. If bars are not visible, they are within the symbol. * *p* < 0.05 and **p < 0.001 versus saline solution tested at the same time of treatments.

The results reported in Figure 4 showed that the new vesicular systems were non-toxic and did not induce skin erythema or irritation. Likely, the interaction of oleic and linoleic acids contained in the self-assembled vesicles were able to induce a perturbation of skin lipid structures, as demonstrated by permeation profiles, but without inducing any damage to the tissue. In detail, the values of erythema index variation (ΔE.I.) remained below the ΔE.I. values corresponding to the treatment of healthy volunteers with the saline solution, used as a control; and in any case below the 4 value.

The in vivo results demonstrated that unsaturated fatty acid vesicles are well tolerated on skin, indicating their possible clinical potential with regards to patient compliance and therapeutic efficacy.

## 4. Conclusions

The obtained results are very encouraging and suggest the use of unsaturated fatty acid vesicles as totally natural drug delivery systems for topical administration of ammonium glycyrrhizinate. The physico-chemical characterization showed that both studied formulations were suitable for topical application in terms of mean size, polydispersity index, surface charge, but above all, both unsaturated fatty acid vesicles were able to contain and to in vitro release the entrapped ammonium glycyrrhizinate. The results of percutaneous permeation studies showed that vesicles based on linoleic and oleic acid were able to overcome the issue related to physico-chemical characteristics of the natural compound. In fact, due to its encapsulation in the unsaturated fatty acid vesicles, ammonium glycyrrhizinate was able to across the stratum corneum with high efficacy compared to its free form. In vitro studies, carried out on a skin cell model, have demonstrated the improved antioxidant activity of ammonium glycyrrhizinate when delivered by unsaturated fatty acids vesicles. This result was due to the probable interaction between nanovesicles and cell membranes, as already demonstrated by our research group for another cell model. Finally, in vivo studies, performed on human volunteers, confirmed the hypothesized cutaneous safety profile of empty vesicular systems consisting exclusively of natural components and highlighting biocompatibility toward skin tissue thanks to the presence of natural fatty acids.

Several research groups treated linoleic acid as a drug and encapsulated it within vesicular and non-vesicular systems, demonstrating a good effect on the skin [15,16,17], but, to the best of our knowledge, no one has ever evaluated the skin effects of linoleic and oleic acid structured in bilayers.

## Figures and Tables

**Table 1:** Lipid composition of unsaturated fatty acid vesicles

Scheme	Lipid Composition ^a^			AG ^b^ (mg/mL)
	Oleic Acid	Linoleic Acid	PL90G	
Blank Formulation A	1	1	0.5	-
Blank Formulation B	1	1	-	-
AG-Formulation A	1	1	0.5	3
AG-Formulation B	1	1	-	3

a: molar ratio; b: ammonium glycyrrhizinate.

**Table 2:** Physico-chemical features of blank and ammonium glycyrrhizinate-loaded unsaturated fatty acid vesicles

Sample	Mean Size (nm)	PdI ^1^	Surface Charge (mV)	DI ^2^	EE (%) ^3^	DL (%) ^4^
Blank Formulation A	189 ± 2	0.20 ± 0.02	−44 ± 1	9.71 ± 0.87	-	-
Blank Formulation B	284 ± 2	0.22 ± 0.03	−42 ± 2	12.73 ± 1.01	-	-
AG ^5^-Formulation A	146 ± 1	0.17 ± 0.01	−50 ± 1	9.55 ± 0.59	80.92 ± 1.03	36.43 ± 0.45
AG-Formulation B	153 ± 3	0.21 ± 0.01	−45 ± 1	10.02 ± 1.00	84.98 ± 1.2	38.08 ± 0.60

1: Polydispersity index; 2: Deformability index; 3: Entrapment efficacy; 4: Drug loading capability; 5: Ammonium glycyrrhizinate).

## Data Availability

The data presented in this study are available on request from the corresponding author. The data are not publicly available due to privacy.
